# Magnetic Particle Plug-Based Assays for Biomarker Analysis

**DOI:** 10.3390/mi7050077

**Published:** 2016-04-26

**Authors:** Chayakom Phurimsak, Mark D. Tarn, Nicole Pamme

**Affiliations:** Department of Chemistry, University of Hull, Cottingham Road, Hull, HU6 7RX, UK; chayakom.p@rmutsb.ac.th (C.P.); m.tarn@hull.ac.uk (M.D.T.)

**Keywords:** C-reactive protein (CRP), progesterone (P4), immunoassays, magnetic particles, magnetism, microfluidics, particle trapping

## Abstract

Conventional immunoassays offer selective and quantitative detection of a number of biomarkers, but are laborious and time-consuming. Magnetic particle-based assays allow easy and rapid selection of analytes, but still suffer from the requirement of tedious multiple reaction and washing steps. Here, we demonstrate the trapping of functionalised magnetic particles within a microchannel for performing rapid immunoassays by flushing consecutive reagent and washing solutions over the trapped particle plug. Three main studies were performed to investigate the potential of the platform for quantitative analysis of biomarkers: (i) a streptavidin-biotin binding assay; (ii) a sandwich assay of the inflammation biomarker, C-reactive protein (CRP); and (iii) detection of the steroid hormone, progesterone (P4), towards a competitive assay. Quantitative analysis with low limits of detection was demonstrated with streptavidin-biotin, while the CRP and P4 assays exhibited the ability to detect clinically relevant analytes, and all assays were completed in only 15 min. These preliminary results show the great potential of the platform for performing rapid, low volume magnetic particle plug-based assays of a range of clinical biomarkers via an exceedingly simple technique.

## 1. Introduction

Enzyme-linked immunosorbent assays (ELISA) are a powerful method of identification and quantification, utilising the specificity of labelled antibodies for their complementary antigens to give a signal (e.g., via fluorescence or chemiluminescence) dependent on the concentration of the latter [1,2]. However, while ELISA offers extremely low limits of detection and selectivity, the process is exceedingly slow, requiring multiple reagent and washing steps that are both laborious and time-consuming. The use of magnetic microparticles as solid supports has become incredibly popular for immunoassays and other applications thanks to their high surface-to-volume ratios, small sizes (0.1–100 µm), the range of functional groups that can be attached to the surfaces (e.g., antibodies, DNA, and chemical groups), and the ability to easily manipulate the particles via an applied magnetic field [3,4]. By employing antibody functionalised magnetic particles, immunoassay time frames can be greatly reduced, with permanent magnets used to enable the separation of antigens from the sample and speeding up the exchange of reaction and washing solutions. Even so, these magnetic particle-based assays still require multiple manual solution changes; hence, despite being faster than conventional ELISAs, they are still somewhat slow and require relatively large volumes of solutions.

The application of microfluidic devices [5,6,7], having channel networks with typical dimensions on the order of 1–100 s of micrometres, provides a number of advantages to immunoassays by reducing diffusion distances, reaction and washing time frames, as well as sample and reagent volumes [8,9,10,11,12]. Integration of magnetic particles with microfluidics thus combines the benefits of both [13,14,15,16], and has yielded great success for on-chip bioanalysis [12,16]. One of the easiest methods of performing techniques such as immunoassays is via the trapping of functionalised microparticles within the microchannel [17,18], before pumping solutions of sample, washing buffer and reagents over the particles. Trapping magnetic particles within a microchannel can easily be achieved by simply applying an external magnetic field that can be generated via a number of sources, including permanent magnets [19,20,21,22,23,24,25,26,27,28,29,30,31,32], integrated microelectromagnets [33,34,35,36,37,38], electromagnets [39,40,41,42,43], and externally magnetisable integrated microstructures [44,45,46,47,48,49].

Most often, this type of setup is used for the separation of target analytes from a sample; as the sample is pumped over the trapped magnetic particles the analytes bind to the functional groups on the particles, after which the particles are washed with buffer solution [13]. However, immunoassays are performed by consecutively flushing sample, reagent and washing solutions over antibody-coated particles, thereby enabling selection of a target analyte from a sample and its subsequent labelling (e.g., with a fluorescent tag) for detection. Such processes have been applied to assays for streptavidin-biotin [38,50], protein A [31,50], mouse IgG [26,29,51], parathyroid hormone [52], interleukin-5 [52], bovine serum albumin (BSA) [42], alkaline phosphatase [53], and glycine [50]. Modifications to this methodology have included the use of segmented flow, in which the consecutive reaction and washing solutions are contained within droplets that are pumped over the trapped particles [53], and the generation of a fluidised bed of magnetic particles to enhance mixing [29]. However, while modifications such as these can yield low detection limits with small sample volumes, it often comes at the cost of greater complexity. Further applications beyond immunoassays have included RNA isolation [54,55], DNA hybridisation [56,57,58] and separation [59], purification of polymerase chain reaction (PCR) products for gene synthesis [60], cell capture for DNA detection [61,62], reaction rate measurements [63], protein digestion [28,64,65,66], and the electrochemical detection of peroxide [48], among others.

**Figure 1:**
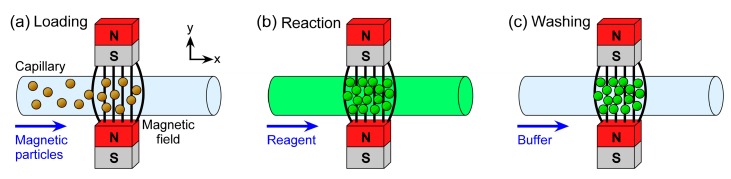
Principle of magnetic particle plug-based assays: (**a**) functionalised magnetic particles are introduced into a microchannel and trapped between two magnets, forming a plug; (**b**) a fluorescently labelled reagent or sample solution is flushed over the particle plug, with the reagent or target analyte binding to the particles; and (**c**) the microchannel is washed with buffer solution, allowing fluorescence detection of the trapped particle plug.

Previously, we have demonstrated the trapping of plugs of magnetic particles in microchannels for performing simultaneous assays on particles featuring different surface functionalities [50]. In order to maintain a simple setup and user-friendliness, the apparatus consists only of a capillary placed between two permanent magnets and connected to a single syringe pump operating in withdrawal mode. Functionalised magnetic particles are first pumped into the microchannel and trapped between the magnets, creating a particle plug that is consecutively exposed to reagent and washing solutions prior to detection of the target analyte using fluorescence (Figure 1). By placing multiple pairs of magnets upstream of each other, three different particle plugs (featuring glycine, protein A, and streptavidin surface groups) were generated for the simultaneous assays. We have also demonstrated how diamagnetic repulsion forces can be employed for performing particle plug-based assays [67,68]. The proof-of-principle work thus far has involved only qualitative assays to test the platform. Here, we investigate the potential for using this simple platform for quantitative analysis towards its application in clinical diagnostics. Three main approaches are described here: (i) the ability to generate a calibration curve and obtain a limit of detection for a streptavidin-biotin binding assay; (ii) the detection in a relevant concentration range of an inflammation and infection biomarker, C-reactive protein (CRP), via a sandwich immunoassay; and (iii) the detection of a clinically relevant steroid hormone, progesterone (P4), at multiple concentrations with a view to competitive assays.

## 2. Materials and Methods

### 2.1. Reagents and Particles

Tris(hydroxymethyl)aminomethane (Tris), 2-(*N*-morpholino)ethanesulfonic acid (MES), *N*-(3-dimethylaminopropyl)-*N*’-ethylcarbodiimide hydrochloride (EDC), *N*-hydroxysuccinimide (NHS), and bovine serum albumin (BSA) were purchased from Sigma-Aldrich (Dorset, UK).

Superparamagnetic particles with a 2.8 µm diameter were purchased from Invitrogen (Paisley, UK) with two different surface functionalities: streptavidin (Dynabeads M-270 Streptavidin) and carboxylic acid (Dynabeads M-270 Carboxylic Acid). Biotin-4-fluorescein (λ_ex_ = 494 nm, λ_em_ = 524 nm) and phosphate buffered saline (PBS) tablets were also purchased from Invitrogen.

Recombinant human C-reactive protein (CRP) and primary CRP antibody (1° anti-CRP; biotinylated mouse anti-human C-reactive protein) were purchased from R&D Systems (Abington, UK). Secondary CRP antibody tagged with a fluorescent label (2° anti-CRP-FITC; polyclonal goat anti-human C-reactive protein conjugated to fluorescein isothiocyanate, λ_ex_ = 495 nm, λ_em_ = 521 nm) was purchased from Abcam (Cambridge, UK) in PBS solution at a stock concentration of 1 mg·mL^−1^. Progesterone labelled with fluorescein isothiocyanate (P4-FITC, 1 mg·mL^−1^ stock solution) and progesterone antibody (anti-P4) were purchased from R&D Systems.

### 2.2. Preparation of Solutions

All solutions were prepared in double-filtered (0.05 µm) high purity water (18.2 MΩ·cm at 25 °C) via an ELGA Option 4 system that fed into an ELGA UHG PS system, both of which were from ELGA Process Water (Marlow, UK).

PBS solution (pH 7.45) was prepared by dissolving a tablet in 1000 mL water, and had BSA added to a concentration of 0.01% *w/v* in order to reduce non-specific binding of reagents and the sticking of magnetic particles to the capillary walls or to each other. Tris buffer (20 mM, pH 8) was prepared by dissolving tris(hydroxymethyl)aminomethane in water, with 0.1% *w/v* BSA added. MES buffer (pH 5) was prepared to a concentration of 25 mM in water.

Fluorescently labelled biotin (biotin-4-fluorescein) was dissolved in PBS solution to a stock concentration of 1 mg·mL^−1^ and protected from light by wrapping the container in aluminium foil. CRP antigen was reconstituted in Tris buffer to a concentration of 200 µg·mL^−1^, as per the manufacturer’s instructions, then diluted in PBS solution to concentrations of 1 µg·mL^−1^ and 10 µg·mL^−1^. Primary CRP antibody (1° anti-CRP) was reconstituted in PBS solution to a concentration of 50 µg·mL^−1^, as per the manufacturer’s instructions, and then further diluted in PBS to 1 µg·mL^−1^. Secondary CRP antibody (2° anti-CRP-FITC) was diluted in PBS to a concentration of 100 µg·mL^−1^. Progesterone antibody (anti-P4) was dissolved in MES buffer (25 mM, pH 5) to a concentration of 1 µg·mL^−1^, while fluorescently labelled progesterone (P4-FITC) was diluted in PBS solution to concentrations of 0.1–100 µg·mL^−1^.

### 2.3. Preparation of Anti-CRP Functionalised Magnetic Particles

Immobilisation of biotinylated primary CRP antibodies (1° anti-CRP) onto streptavidin functionalised magnetic particles (Dynabeads M-270 Streptavidin) was achieved via the streptavidin-biotin interaction, as previously reported [69,70]. Briefly, 10 µL of stock particle suspension (6.5 × 10^8^ particles·mL^−1^) was added to a 1.5 mL microcentrifuge tube (VWR, Leicester, UK), followed by 200 µL of 1° anti-CRP solution at a concentration of 10 µg·mL^−1^, and incubated for 15 min with slow tilt rotation in order for the biotinylated antibodies to bind to the streptavidin-coated particles. The particles were then washed three times using the following procedure.

The particles were pulled to the side of the tube via an external magnet and the supernatant removed using a pipette. PBS solution (1000 μL) was added to the tube, which was vortexed for 20 s to resuspend the particles. This washing process was repeated twice more, and the particles finally resuspended in PBS buffer solution.

### 2.4. Preparation of Anti-P4 Functionalised Magnetic Particles

Immobilisation of progesterone antibody (anti-P4) onto carboxylic acid functionalised magnetic particles (Dynabeads M-270 Carboxylic Acid) was achieved via amide bond formation between the carboxylic acid groups of the particles and the primary amine groups of the antibodies. The procedure was performed as per the manufacturer’s instructions for a two-step coating procedure [71]. The first step of the process involved the “activation” of the magnetic particles with a carbodiimide (EDC) and *N*-hydroxysuccinimide (NHS). One hundred microlitres of stock particle suspension (2 × 10^9^ particles·mL^−1^) was added to a microcentrifuge tube and washed twice, as described previously, with 100 µL of MES buffer (25 mM, pH 5). Immediately prior to use, a 50 mg·mL^−1^ solution of EDC was prepared in cold MES buffer, while a 50 mg·mL^−1^ solution of NHS was also prepared in MES buffer. The supernatant of the particle suspension was removed, and 50 µL of EDC solution and 50 µL of NHS solution were added to the magnetic particles. The suspension was mixed via a vortexer and allowed to incubate with slow tilt rotation at room temperature for 30 min.

Following incubation, the particles were washed twice with 100 µL of MES buffer. The supernatant was removed, and 60 µL of anti-P4 (1 µg·mL^−1^) in MES buffer was added, followed by a further 40 µL of MES buffer. The mixture was incubated for 2 h at 20 °C, then the particles were washed four times with PBS solution (pH 7.45) and finally resuspended in PBS solution.

### 2.5. Instrumental Setup

**Figure 2:**
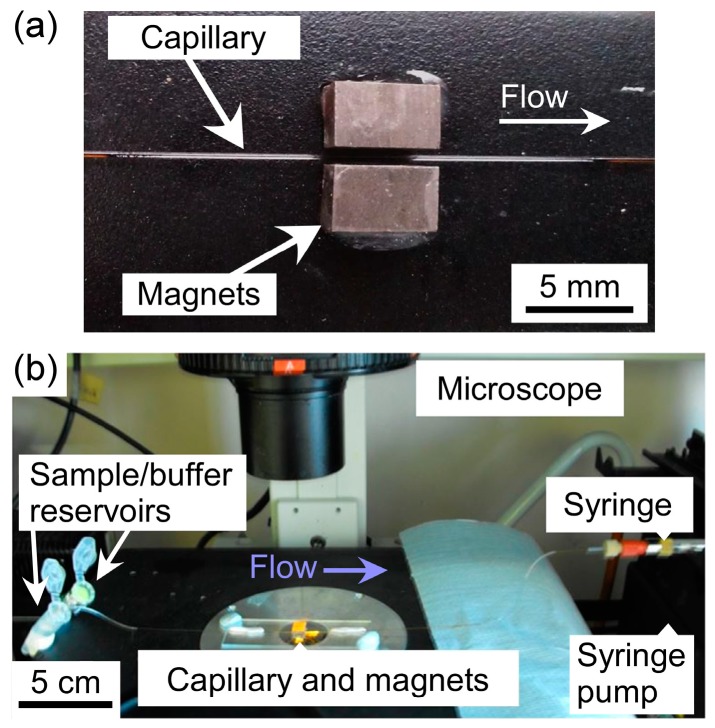
Setup of the microfluidic device: (**a**) photograph of a fused silica capillary located in the 1 mm gap between two 4 × 4 × 6 mm^3^ NdFeB magnets that were fixed to a glass microscope slide; and (**b**) photograph of the glass microscope slide, holding the capillary and magnets, on the sample stage of an inverted fluorescence microscope. Samples, reagents and buffer solutions were introduced into the capillary from reservoirs via a syringe pump in withdrawal mode.

Two rectangular neodymium-iron-boron (NdFeB) magnets (4 × 4 × 6 mm^3^, Magnet Sales, Swindon, UK) were glued onto a glass microscope slide (6 × 2.5 cm^2^) using Araldite Rapid epoxy resin (RS Components, Northants, UK), such that their opposing poles were facing and there was a 1 mm gap between them. A 10 cm long piece of fused silica capillary (150 µm ID (Inner Diameter), 363 µm OD (Outer Diameter), CM Scientific, Silsden, UK) had a section of its polyimide coating burned away with a lighter and wiped with a soft tissue to create a region for visualisation of trapped particles. The capillary was then placed between the pair of magnets (Figure 2a) and held in place using Blu-Tack (Bostick, UK). The two ends of the capillary were connected to Tygon tubing (254 µm ID, 762 µm OD, Cole-Parmer, London, UK), with one piece of tubing interfaced to a syringe on a syringe pump (PHD 22/2000, Harvard Apparatus, Kent, UK) and the other piece of tubing dipped into a microcentrifuge tube, acting as a reservoir, containing sample or buffer solution.

Solutions were drawn through the capillary from the sample/buffer reservoir via negative pressure from the syringe pump operating in withdrawal mode. The glass slide holding the magnets and capillary setup was situated on the sample stage of an inverted fluorescence microscope (TE-2000U, Nikon, Surrey, UK) (Figure 2b). Images were captured via a cooled CCD (charge-coupled device) camera (QImaging Retiga-EXL, Media Cybernetics, Buckinghamshire, UK) and Image-Pro Plus 6 software (Media Cybernetics, Buckinghamshire, UK). Such images were analysed using ImageJ software (US National Institutes of Health, Bethesda, MD, USA).

### 2.6. Experimental Procedures

#### 2.6.1. Capillary-Based Particle Trapping and Reactions

Prior to performing an experiment, the capillary was cleaned and pre-treated by flushing consecutively with ethanol, water and PBS solution. Following this, the inlet tubing connected to the capillary was dipped into a suspension of magnetic particles in a microcentrifuge tube and negative pressure applied via the syringe pump to draw the particle suspension through the capillary. After a certain time frame, the syringe pump was stopped and the flow allowed to come to a halt (~30 s in order to prevent air from entering the system during solution exchange) before the inlet tubing was removed from the particle suspension vial and placed into a vial of PBS solution. PBS was then drawn through the capillary for several minutes to ensure that all particles within the capillary would reach the region between the two magnets to form a plug of particles. Characterisation of the particle plugs was performed at this stage by taking photographs via the microscope and CCD camera, and analysing the images with ImageJ software to determine the area of the plugs.

To perform a reaction on the particle plug, once the particle suspension had been introduced into the capillary, the microcentrifuge tube was exchanged for one containing a reagent solution, which was pumped through the capillary for several minutes such that it was allowed to wash over the particle plug, before the pump was again stopped. When the flow had stopped, the inlet tubing was placed in a vial of PBS solution, which was pumped over the particle plug to wash away any unbound material. The CRP assay, being a two-step sandwich assay, required a second reaction following the first. Finally, fluorescence images were taken of the particle plug and the fluorescence intensity of the plugs measured via ImageJ. Analysis was performed manually by drawing a small box inside the image of the particle plug, determining the maximum greyscale value (as a measure of fluorescence intensity), and deducting an average background intensity. This process was repeated for several regions inside the particle plug to provide average of the maximum fluorescence intensities.

#### 2.6.2. Formation and Characterisation of Magnetic Particle Plugs

Prior to performing reactions, the formation of the particle plugs was characterised based on the applied flow rate and the particle concentration. A suspension of Dynabeads M-270 Carboxylic Acid particles in PBS buffer was pumped into the capillary for 90 s, then the solution swapped to PBS which was pumped through the capillary for a further 10 min. Images of the forming particle plug were collected every minute and experiments were repeated three times. Flow rates of 180–300 µL·h^−1^ (equivalent to linear velocities of 2.8–4.7 mm·s^−1^) and particle concentrations of 1 × 10^6^ to 2 × 10^7^ particles·mL^−1^ were studied.

#### 2.6.3. Streptavidin-Biotin Assay

In order to test and optimise reactions on the setup, a streptavidin-biotin binding assay was investigated. Streptavidin functionalised magnetic particles (Dynabeads M-270 Streptavidin) in PBS solution (1 × 10^7^ particles·mL^−1^) were pumped through the capillary for 2 min at a flow rate of 300 µL·h^−1^ (4.7 mm·s^−1^) to form a particle plug between the NdFeB magnets. The particle suspension vial was exchanged for one containing biotin-4-fluorescein solution, which was flushed over the trapped particle plug at 300 µL·h^−1^ for 3 min, then the sample vial was exchanged again for PBS solution. The PBS solution was drawn through the capillary for 3 min at 300 µL·h^−1^ to wash the particle plug, whose fluorescence intensity was then measured. The concentration of biotin-4-fluorescein was varied between 0.1–5 µg·mL^−1^. The effect of exposure time during the capture of fluorescence images was also studied using the streptavidin-biotin reaction for optimisation.

#### 2.6.4. C-Reactive Protein (CRP) Assay

Magnetic particles featuring surface-bound primary CRP antibodies (1° anti-CRP), prepared as described in Section 2.3, in PBS solution (1 × 10^7^ particles·mL^−1^) were introduced into the capillary at a flow rate of 300 µL·h^−1^ (4.7 mm·s^−1^) for 2 min in order to form the particle plug between the two magnets. The particle suspension tube was replaced with a tube containing CRP solution (1 or 10 µg·mL^−1^), which was pumped over the particle plug for 3 min at the same flow rate. Fluorescently tagged secondary antibody (2° anti-CRP-FITC) solution (100 µg·mL^−1^) was then flushed over the particle plug for 3 min at 300 µL·h^−1^, and the plug was finally washed with PBS solution for 5 min prior to fluorescence measurement of the particles.

#### 2.6.5. Progesterone (P4) Assay

Magnetic particles functionalised with progesterone antibody (anti-P4), prepared as described in Section 2.4, in PBS solution (1 × 10^7^ particles·mL^−1^) were pumped into the capillary at a flow rate of 300 µL·h^−1^ (4.7 mm·s^−1^) for 2 min for plug formation between the magnets. A solution of fluorescently labelled progesterone (P4-FITC), whose concentration was varied from 0.1–100 µg·mL^−1^, was subsequently flushed over the trapped plug at 300 µL·h^−1^ for 3 min, before washing the plug with PBS solution for 3 min to remove any unbound P4-FITC. Fluorescence analysis was then performed on the trapped magnetic particle plug.

## 3. Results and Discussion

### 3.1. Formation and Characterisation of Magnetic Particle Plugs

**Figure 3:**
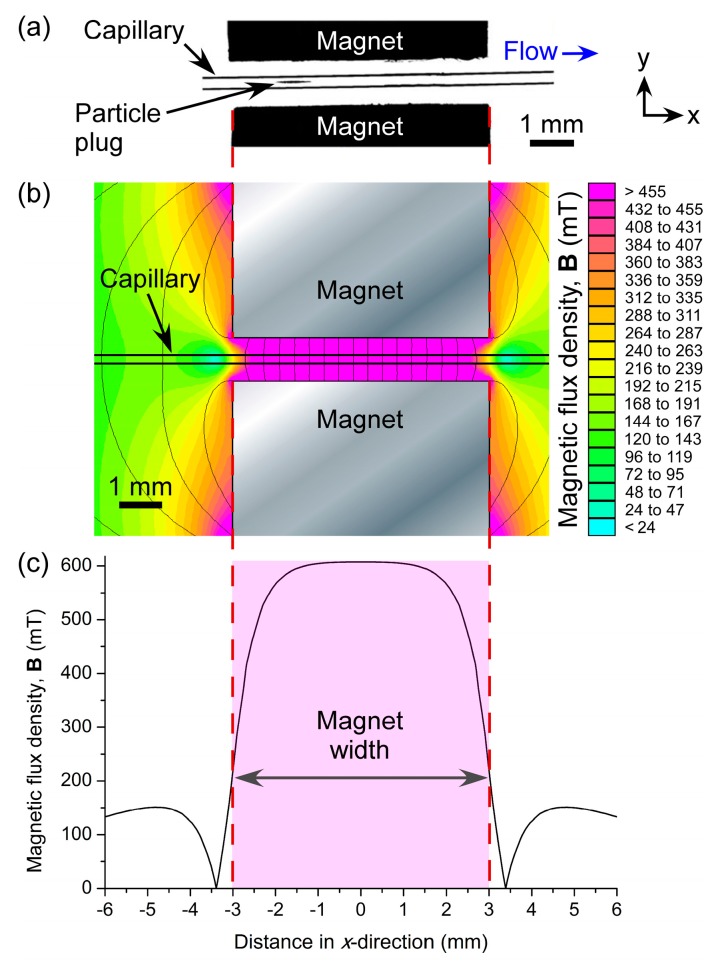
(**a**) Photograph of a plug of magnetic particles trapped between two NdFeB magnets in a capillary. (**b**) Simulation of the magnetic flux density (**B**) across the microfluidic channel, modelled using FEMM software. (**c**) Plot of the magnetic flux density along the length of the capillary (*x*-direction) between the two magnets.

When particle suspensions were introduced into the fused silica capillary via negative pressure, they flowed freely through the tube until they approached the two magnets. At this point, they became trapped in the field between the magnets and began to form a plug of particles that grew larger as particles continued to be introduced into the 150 µm ID capillary. The particles remained stationary as they were trapped in the field, as opposed to the continuously recirculating plugs observed when diamagnetic particles are trapped in a magnetic fluid [67,68]. The location of particle plug formation was *x* ≈ 1.7 mm from the centre (*x* = 0 mm) of the magnets (Figure 3a). In order to investigate this further, the magnetic field was modelled in FEMM 4.2 software (Figure 3b). The resultant simulations showed that the region of highest magnetic flux density (vectors not shown) was located between *x* ≈ −1.5 mm and *x* ≈ 1.5 mm (Figure 3c), hence the location of particle trapping was as predicted.

As stated above, the particles were able to flow freely through the capillary. No evidence of sedimentation due to gravity was observed, with the theoretical forces on the particles due to gravity being calculated as 68 femtonewtons (fN), yielding a sedimentation velocity of 2.6 µm·s^−1^. This value was negligible compared to the minimal linear flow rate of 2.8 mm·s^−1^ (at a volumetric flow rate of 180 µL·h^−1^) in the capillary. Furthermore, inertial lift forces may also have helped to prevent particles from settling against the capillary wall while in flow. These observations also supported those from our previous work [50,67,68]. The sticking of particles to the capillary walls was minimal, with BSA added to solutions to prevent this from occurring, and was found not to interfere with experiments.

Prior to performing assays on trapped particles, two parameters affecting plug formation were investigated, namely the applied flow rate and the particle concentration, towards optimisation in terms of rapid formation of a plug deemed large enough for yielding suitable fluorescence signals. Carboxylic acid functionalised magnetic particles (2.8 µm) in PBS solution (pH 7.45) were employed for these tests.

#### 3.1.1. Effect of Flow Rate

The applied flow rate is an important parameter since high flow rates would allow the collection of more particles in the trap in a shorter period of time. However, too high a flow rate could lead to particles escaping the trap when the hydrodynamic forces dominate the magnetic forces. Here, particle suspension (1 × 10^6^ particles·mL^−1^) was introduced into the capillary at a flow rate of 5 µL·min^−1^ for 90 s. The sample vial was then exchanged for one containing PBS solution, which was pumped through the capillary at flow rates of 180, 240 and 300 µL·h^−1^ (equivalent to linear velocities of 2.8, 3.8 and 4.7 mm·s^−1^, respectively) in order to determine the effect of flow rate on plug formation. Images of the plugs were captured every minute for 10 min.

**Figure 4:**
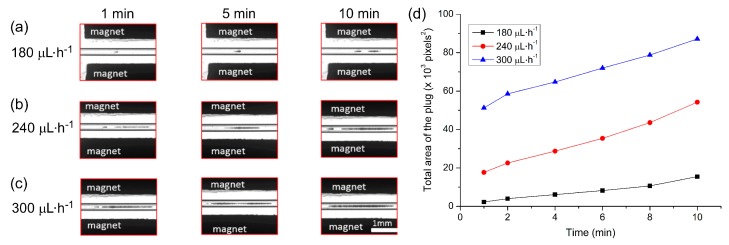
The effect of flow rate on magnetic particle plug formation. (**a**–**c**) Photographs of plug formation at time points of 1, 5 and 10 min for flow rates of: (**a**) 180 µL·h^−1^; (**b**) 240 µL·h^−1^; and (**c**) 300 µL·h^−1^. (**d**) Plot of measured plug sizes over time at the three different flow rates. Each pixel was approximately equivalent to an area of 5.6 µm^2^.

The build-up of the particle plugs can be seen in Figure 4a–c, which shows the size of the plugs at time frames of 1 min, 5 min, and 10 min after initialising the flow. The total area occupied by a particle plug (units of pixels^2^, with one square pixel comprising approximately 5.6 µm^2^) was measured using ImageJ and the results are plotted in Figure 4d, which clearly demonstrates that faster flow rates yielded larger plugs of trapped particles. Interestingly, however, it appeared that following the first minute of trapping, the plugs actually grew at similar rates at each of the three flow rates. This may have been due to the build-up of the plugs in three dimensions within the capillary while only a 2D image could be taken for analysis, a parameter that could be explored in future work. Importantly, even at the highest flow rate tested of 300 µL·h^−1^ (4.7 mm·s^−1^), 100% trapping efficiency was achieved. Hence, this flow rate was employed in all subsequent experiments to yield the rapid formation of large plugs with no loss of particles.

#### 3.1.2. Effect of Particle Concentration

The effect of particle concentration on plug formation was investigated by pumping the magnetic particles through the capillary at concentrations of 5 × 10^6^, 1 × 10^7^ and 2 × 10^7^ particles·mL^−1^ for 90 s at 300 µL·h^−1^. PBS solution was then flushed through the capillary for 10 min at the same flow rate, with photographs taken of the growing particle plug every minute.

**Figure 5:**
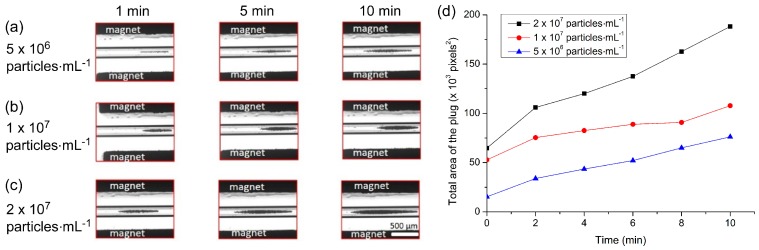
The effect of particle concentration on plug formation. (**a**–**c**) Photographs of plug formation at time points of 1, 5 and 10 min for particle concentrations of: (**a**) 5 × 10^6^ particles·mL^−1^; (**b**) 1 × 10^7^ particles·mL^−1^; and (**c**) 2 × 10^7^ particles·mL^−1^. (**d**) Plot of measured plug sizes over time at the three different particle concentrations. Each pixel was approximately equivalent to an area of 5.6 µm^2^.

Images of the trapped particle plugs for each concentration can be seen in Figure 5a–c at 1, 5 and 10 min after starting the washing step. As expected, higher particle concentrations yielded larger plugs in shorter times. The total area of each plug was analysed using ImageJ software and the results are plotted in Figure 5d. Again, the rates of plug formation were actually largely quite similar in each case (from a 2D viewpoint at least), but nonetheless reaffirmed that the higher the particle concentration was, the larger the plug that was formed within a certain time frame. However, the plug formed at higher concentrations was a lot more spread out across the capillary (in the *x*-direction), hence a concentration of 1 × 10^7^ particles·mL^−1^ was employed for subsequent experiments in order to form a fairly large plug with a better defined shape.

### 3.2. Streptavidin-Biotin Assay

In order to test the setup for performing reactions on particle plugs, proof-of-principle assays based on the streptavidin-biotin interaction were performed. While we have previously demonstrated a streptavidin-biotin binding assay on a magnetic particle plug [50], the tests were only qualitative. Here, we investigated the ability to produce calibration curves of biotin concentrations using the magnetic trapping platform.

**Figure 6:**
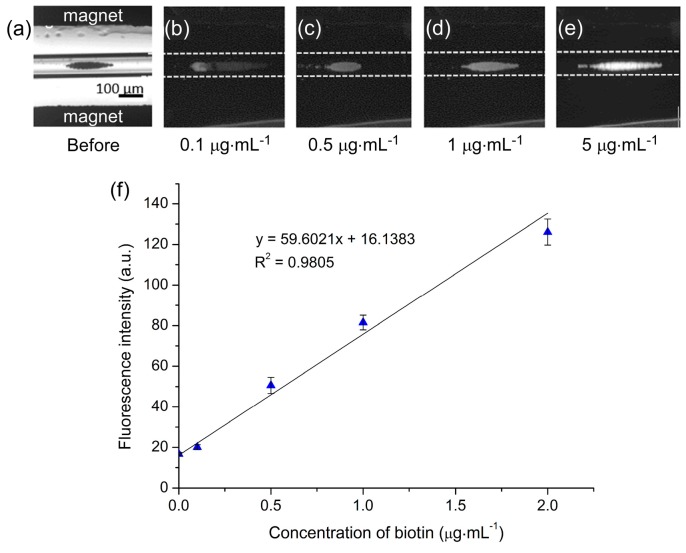
Streptavidin-biotin assays performed by flushing a solution of fluorescently labelled biotin over a trapped plug of streptavidin functionalised magnetic particles. (**a**) Bright-field image of the trapped particle plug. (**b**–**e**) Fluorescence images of streptavidin particle plugs exposed to varying concentrations of biotin: (**b**) 0.1 µg·mL^−1^; (**c**) 0.5 µg·mL^−1^; (**d**) 1 µg·mL^−1^; and (**e**) 5 µg·mL^−1^. (**f**) Calibration graph of particle plug fluorescence intensities exposed to a range of fluorescently labelled biotin concentrations.

Dynabeads M-270 Streptavidin particles (1 × 10^7^ particles·mL^−1^) were pumped into the capillary at a flow rate of 300 µL·h^−1^ for 2 min to generate the particle plug between the magnets. This was followed by a solution of biotin-4-fluorescein at 300 µL·h^−1^ for 3 min, and finally a solution of PBS for 3 min in order to wash the particle plug. A range of biotin-4-fluorescein concentrations (0.1–5 µg·mL^−1^) were tested, and photographs of particle plugs exposed to each of these concentrations are shown in Figure 6a–e. Clearly, as the concentration of biotin was increased the fluorescence intensity of the particle plug also increased, indicating successful binding of the fluorescent biotin to the streptavidin-coated particles.

In order to optimise the platform further for quantitative analysis, the effect of CCD camera exposure time was investigated alongside the ability to generate calibration curves. Multiple concentrations of biotin (0.1–5 µg·mL^−1^) were flushed over particle plugs and analysed using a range of CCD camera exposure times (0.1–0.6 s). The resultant plots are shown in Figure S1a and demonstrate typical dose–response curves, with the fluorescence intensity first increasing sharply as the biotin concentration increased before reaching a plateau at ~2 µg·mL^−1^ as the number of streptavidin binding sites on the particles diminished. The polystyrene matrix of the magnetic particles exhibited auto-fluorescence, hence the non-zero fluorescence intensity even in the absence of biotin. Due to the plateau above 2 µg·mL^−1^, the curves were re-plotted to show the linear responses between 0.1–2 µg·mL^−1^ (Figure S1b), demonstrating the suitability of the platform for quantitative analysis.

Clearly, as the CCD camera exposure time was increased the measured fluorescence intensities also increased, as expected since more of the fluorescence light was allowed to enter the CCD. However, while higher exposure times yielded greater intensities, they also exhibited poorer coefficients of determination (*R^2^*) as demonstrated in Figure S1b. Based on this, the results obtained with the 0.3 s exposure time were selected as being the optimum, with the calibration curve (Figure 6f) yielding a limit of detection (LOD) of 40 ng·mL^−1^ and a limit of quantification (LOQ) of 134 ng·mL^−1^ for fluorescently labelled biotin.

These results demonstrated the potential of the platform for quantitative analysis with low limits of detection in a fast time frame (<10 min), while consuming only 15 µL of sample/reagent. Furthermore, some aspects could be optimised further, such as the reaction times, which would lead to shorter procedural times and lower reagent consumption, and the washing times. The detection method could also be improved to increase the coefficient of determination while reducing the limits of detection further. While these will be investigated further in later studies, the promising initial results prompted further studies with more clinically relevant biomarkers towards the use of the platform as a diagnostic tool.

### 3.3. C-Reactive Protein (CRP) Assay

The first clinically relevant biomarker tested using the magnetic plug platform was C-reactive protein (CRP) [72,73,74,75]. CRP is an acute phase reactant present in blood whose levels increase dramatically, up to 1000-fold, in response to inflammation, cell damage or tissue injury, hence its monitoring in a clinical setting for infections and inflammation. Normal levels of CRP in serum are considered to be 1–10 µg·mL^−1^, with levels of 10–40 µg·mL^−1^ suggesting viral infection or mild inflammation while levels of 40–200 µg·mL^−1^ indicate active inflammation or bacterial infection. Chronic minor elevations in CRP levels may also be an indicator for cardiovascular disease (CVD), hence so-called high-sensitivity CRP (hs-CRP) testing is performed to monitor levels over time (<1µg·mL^−1^ = low CVD risk; 1–3 µg·mL^−1^ = medium risk, >3 µg·mL^−1^ = high risk) [76,77,78]. Due to its clinical relevance and the detection levels required, CRP was deemed to be an excellent choice for testing the ability to perform sandwich enzyme-linked immunosorbent (ELISA) assays using the magnetic particle plug platform.

Magnetic particles functionalised with 1° anti-CRP (1 × 10^7^ particles·mL^−1^) were introduced into the capillary at 300 µL·h^−1^ for 2 min for magnetic particle plug formation. This was followed by a solution of CRP for 3 min, allowing the CRP analyte to bind to the antibody-coated particles, before being flushed with a solution of 2° anti-FITC (100 µg·mL^−1^) for 3 min that fluorescently labelled the captured CRP analyte. Finally, the particle plug was washed with PBS solution for 5 min and fluorescence images recorded for analysis.

**Figure 7:**
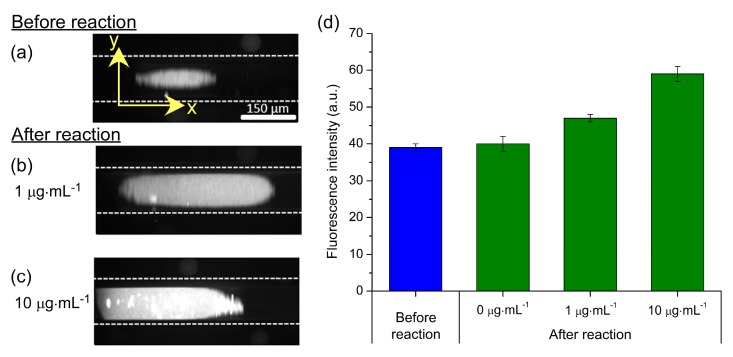
Results obtained via a magnetic particle plug-based sandwich assay for C-reactive protein (CRP). (**a**) Fluorescence image of a particle plug prior to the CRP assay, demonstrating the auto-fluorescence of the polystyrene-based particles. Magnetic particles were functionalised with primary CRP antibodies (1° anti-CRP). (**b**) Fluorescence exhibited by a particle plug after exposure to 1 µg·mL^−1^ CRP and subsequent labelling with fluorescently tagged secondary CRP antibody (2° anti-CRP-FITC; 100 µg·mL^−1^); and (**c**) after exposure to 10 µg·mL^−1^ CRP and labelling with 2° anti-CRP-FITC (100 µg·mL^−1^). (**d**) Plot of fluorescence intensities of the particle plugs at varying concentrations of CRP.

Fluorescence images of particle plugs are shown in Figure 7a–c. Figure 7a demonstrates the auto-fluorescence of the particles prior to a reaction being performed, while Figure 7b,c show the effects of exposure to CRP concentrations of 1 µg·mL^−1^ and 10 µg·mL^−1^, respectively, followed by reaction with the 2° anti-CRP-FITC (100 µg·mL^−1^). The photographs clearly show an increase in fluorescence intensity with increasing CRP concentration, and the fluorescence intensity of the particle plugs are plotted in Figure 7d. While a full range of CRP standards was not tested, it is nonetheless clear that clinically relevant concentrations of CRP (>10 µg·mL^−1^ for inflammation and infection; 1–10 µg·mL^−1^ for CVD monitoring) could be distinguished from each other and from the unreacted particles. Furthermore, negative controls were performed to ensure that unspecific binding of reagents to the particles did not occur. Here, streptavidin-coated magnetic particles, having not undergone the 1° anti-CRP functionalisation step, were trapped in the capillary and flushed with CRP (10 µg·mL^−1^) and 2° anti-CRP-FITC (100 µg·mL^−1^). Image analysis showed no increase in fluorescence that confirmed a lack of unspecific binding, as previously demonstrated [69,70].

These results demonstrated the ability to perform two-step sandwich immunoassays for clinically relevant biomarkers. Further investigation will be required to determine whether a suitable calibration curve can be generated in the 1–10 µg·mL^−1^ region for hs-CRP testing, but its use for the determination of inflammation and infection, requiring less sensitivity, appears easily achievable. The 3 min analyte capture (CRP) and labelling (2° anti-CRP-FITC) steps at 300 µL·h^−1^ resulted in the consumption of 15 µL of both the sample and the relatively expensive labelling reagent. While already a low volume of each, this could be further reduced by optimising the reaction times. Furthermore, the total time of the magnetic plug-based was <15 min; far faster compared to conventional off-chip magnetic particle-based assays (50 min) and traditional ELISA testing (80 min) [69]. Future work will involve generation of a full calibration range for both conventional CRP and hs-CRP concentration ranges, and the analysis of real serum samples.

### 3.4. Progesterone (P4) Assay

Having established that the magnetic particle plug-based platform could be used for sandwich ELISAs, we next investigated the potential of the system towards achieving competitive ELISAs of clinically relevant biomarkers. Initial tests for this involved the detection of fluorescently labelled progesterone (P4-FITC). Progesterone (P4) is a steroid hormone that plays an important role in the menstrual cycle and pregnancy, being secreted to help prepare the uterus for pregnancy and, following conception, to ensure development of the embryo [79,80,81,82]. Thus, the monitoring of P4 can be used to determine the time at which fertility is highest, for the diagnosis of early pregnancy, to check for the risk or occurrence of miscarriage, and for the detection of adrenal or ovarian cancer. Levels of progesterone are typically less than 1 ng·mL^-1^ pre-ovulation, increasing to 5–20 ng·mL^-1^ mid-menstruation cycle, 11.2–90.0 ng·mL^-1^ in the 1st trimester of pregnancy, 25.6–89.4 ng·mL^-1^ in the 2nd trimester, and 48–150 to ≥300 ng·mL^−1^ in the 3rd trimester [82], with levels being present up to a maximum of 1 g·mL^−1^ in serum [83].

**Figure 8:**
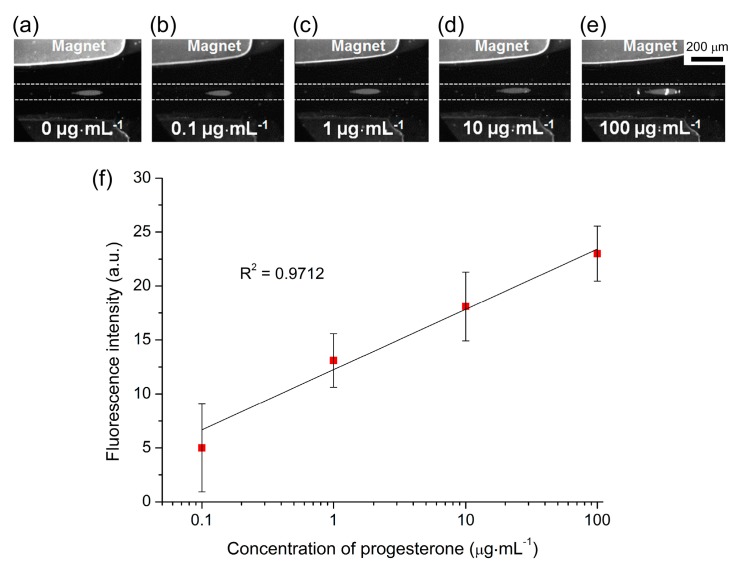
Results obtained for a progesterone (P4) assay, achieved by flushing P4-FITC over a trapped plug of anti-P4 functionalised magnetic particles. (**a**–**e**). Fluorescence images of particle plugs with increasing P4-FITC concentrations. (**f**) Plot of background-corrected particle plug fluorescence intensities at different concentrations (shown on a logarithmic scale) of P4-FITC.

Preliminary tests were performed on the magnetic plug platform by first introducing anti-P4 functionalised magnetic particles (1 × 10^7^ particles·mL^−1^) into the capillary at 300 L·h^−1^ for 2 min to form the plug, before flushing the plug with P4-FITC for 3 min, and finally washing the plug for 3 min with PBS solution. A range of P4-FITC concentrations were tested, from 0.1–100 g·mL^−1^, and the fluorescence intensity of the particle plug was measured at each concentration. Due to the proof-of-principle nature of this study, the levels of P4 tested covered the upper end of the P4 concentration range typically found during the 3rd trimester and the maximum level found in blood (1 g·mL^−1^). Figure 8a–e shows fluorescence images at each of the concentrations. The fluorescence signals measured at each P4-FITC concentration are shown in Figure S2 in the Supplementary Material, demonstrating a typical dose–response curve with an initially rapid increase in signal intensity as the concentration increased, before reaching a plateau as the number of active sites on the magnetic particles was diminished. Plotting the fluorescence intensity against the logarithm of the P4-FITC concentration (background corrected) yielded a linear response over this wide calibration range (Figure 8f). However, the standard deviations of the results were quite large in this case, which may have been caused in part by the relatively low magnification employed for image capture (see the photographs in Figure 8), which would affect the signal intensity. This could be addressed by capturing images of the particle plugs at a higher magnification or by employing a different detection technique. Negative controls were also performed by flushing P4-FITC over a plug of carboxylic acid functionalised particles (*i.e.*, particles which had not had anti-P4 conjugated to them) for 1 h, which thereafter exhibited no fluorescence and so confirmed no issues with unspecific binding.

These preliminary studies show the feasibility of performing competitive ELISAs for biomarkers, such as progesterone hormone, in 10 min and using only 10 µL of sample, though clearly further work is required in order to develop the method into a viable platform for clinical hormone analysis. The next steps towards this goal will be the introduction of unlabelled P4 at varying concentrations alongside the P4-FITC in order to perform actual competitive assays, while the limit of detection and linear range will be explored at the more clinically relevant levels of 1 ng·mL^−1^ to 1 µg·mL^−1^. This would then lead to the testing of real serum samples.

## 4. Outlook

We have performed preliminary studies to establish the feasibility of applying the magnetic particle plug-based platform for clinically relevant bioassays. Characterisation of particle plug formation was performed in order to generate large particle plugs in a short timeframe, and three types of assay systems were investigated: streptavidin-biotin binding assays for evaluation of the platform for quantitative assays, C-reactive protein (CRP) assays for testing the ability to perform sandwich ELISAs of a biomarker in a clinically relevant concentration range, and fluorescently-labelled progesterone (P4-FITC) assays with a view to competitive ELISAs for hormone analysis. While optimisation is still required, these tests show great promise for quantitative analysis of a variety of biomarkers. In particular, the CRP assay, which can already be applied in a relevant concentration range, requires only a full calibration curve to be generated prior to analysis of real samples. The P4 analysis requires more work; while the assay mechanism operates as required and differences in concentration can be detected using the relatively high concentrations tested, the limits of detection need to be established and a calibration curve generated using fluorescently labelled and unlabelled P4 in a clinically relevant range before real serum analysis will be possible. In the case of both analyses, however, testing of robustness of the platform will also be required, including tests of inter-day and inter-chip variability.

The magnetic particle plug-based platform represents an extremely simple setup, requiring only a capillary, two NdFeB magnets, a syringe pump, and a detection system. It also brings several other advantages such as the speed with which assays can be performed. Each step of the process (particle loading, reagent addition, washing) took only 2–3 min each, meaning that total times for each assay was <15 min. Using other methods of performing CRP assays for comparison [69], typical ELISAs will take ~80 min and off-chip magnetic particle-based assays require ~50 min due to the multiple manual reaction and washing steps that are both time-consuming and labour-intensive. Hence, the on-chip platform here represents a far faster approach, while typically using only 15 µL of sample and 15 µL of the expensive labelling reagents compared to the hundreds of microliters of reagents used in ELISA and conventional magnetic particle assays (although ELISA only requires 5 µL of sample). However, further optimisation of reagent and washing times could lead to further reductions in time frames for the magnetic particle plug-based assays, which would in turn result in the use of lower volumes of samples and reagents.

Integrated microelectromagnets could potentially be employed as part of the system to enable finer control of the magnetic field and the ability to switch the field on-and-off as required, as has been demonstrated previously [33,34,35,36,37,38]. However, adding such components would increase the complexity of the setup; an aspect we are trying to avoid in our goal of developing a very simple, robust, user-friendly platform.

While the amount of time allowed for the reaction and washing steps is one method of reducing overall time frames, another would be to decrease the time required to switch between the different solutions (magnetic particle suspension, reagent solutions, washing buffer) being introduced into the microchannel. In the current system, the syringe pump was paused and the flow allowed to come to a stop before manually moving the inlet tubing from one sample or buffer reservoir to the next, with care taken not to allow introduction of air into the capillary during the exchange. These steps could be made far faster, and without the worry of introducing air bubbles, by employing a multi-port valve that allows simultaneous connection of each reservoir to the inlet tubing (e.g., the V-240 6-Way Selection Valve from IDEX Health & Science [84]). Alternatively, a moving array of microvial reservoirs at the capillary inlet could be employed, as has been successfully implemented for sample introduction in microfluidic capillary electrophoresis [85]. In addition, by writing a simple program for controlling such a valve or microarray system and the syringe pump, it would be very easy to automate the various steps of the assays. This would also help to enable multiplexed assays by easily allowing the generation of multiple particle plugs having different surface functionalities for various analytes, as we have demonstrated previously [50,68].

Furthermore, while current detection was achieved using a standard fluorescence microscope, which brings with it an associated bulk and expense, recent advances in miniaturised fluorescence detection systems could conceivably be applied to this platform to yield a far more compact and portable system [86,87,88]. While fluorescence detection was employed in the experiments described here, this could be replaced with a chemiluminescence setup by exchanging the fluorescent tags on the antibodies/antigens for a suitable enzyme, e.g., horseradish peroxidase (HRP), and the washing of the particle plug with a solution of a chemiluminescent substrate solution. This would also reduce the detection setup to a photomultiplier tube (PMT) without the need for a light source.

Clearly, there are a number of steps to be completed before a true analysis platform can be established, and the improvements suggested above would enable a faster, more sensitive, and more compact assay system that uses only small volumes of samples and reagents while requiring minimal manual steps. However, this represents a longer-term vision for the system. Nonetheless, the results described here have demonstrated the use of the miniaturised magnetic particle plug-based assay platform for the detection of several analytes at varying concentrations, showing great potential for fast, low volume sandwich ELISAs and competitive ELISAs for a range of clinically relevant biomarkers.

## 5. Conclusions

We have demonstrated a fast, low volume assay platform in which functionalised magnetic particles are introduced into a microchannel and trapped as a plug between two permanent magnets, allowing their subsequent exposure to consecutive reagent and washing solutions, followed by fluorescence analysis of the particle plug. The formation of the particle plug was characterised and the ability to perform quantitative analysis determined using a streptavidin-biotin binding assay (LOD = 40 ng·mL^−1^). The capacity to detect clinically relevant biomarkers was explored using the inflammation marker, C-reactive protein (CRP), in a sandwich assay, and the steroid hormone, progesterone (P4), in a binding assay with a view to competitive ELISAs. Assays were achieved in less than 15 min, a significant reduction in time compared to conventional procedures, and used only 10–15 µL each of samples and reagents. This shows the potential of the platform for the rapid detection of a range of biomarkers, and future work will involve further optimisation of the setup and the procedure for the analysis of real samples.

## Supplementary Materials

The following are available online at http://www.mdpi.com/2072-666X/7/5/77/s1, Figure S1: Optimisation of CCD camera exposure time. The fluorescence intensity of streptavidin functionalised particle plugs is plotted against the concentration of fluorescently labelled biotin at different CCD exposure times (0.1–0.6 s): (a) biotin concentrations of 0.1–5 μg·mL^−1^, demonstrating a typical dose-response curve; and (b) linear range plotted for 0.1–2 g·mL^−1^ biotin concentration, Figure S2: Fluorescently labelled progesterone (P4-FITC) assay. Magnetic particles functionalised with anti-P4 were exposed to different concentrations of P4-FITC and the resultant fluorescence intensities were measured. The plot follows a typical dose–response curve.
