# Supplementary material for: Magnetic Particle Plug-Based Assays for Biomarker Analysis
Source: Micromachines (Basel). 2016 Apr 26;7(5):77. doi: 10.3390/mi7050077 (PMC6190463; doi:10.3390/mi7050077)
Supplement: Supplementary file 1 [file micromachines-07-00077-s001.pdf]

# Supplementary Material: Magnetic Particle Plug-Based Assays for Biomarker Analysis

**Chayakom Phurimsak, Mark D. Tarn and Nicole Pamme**

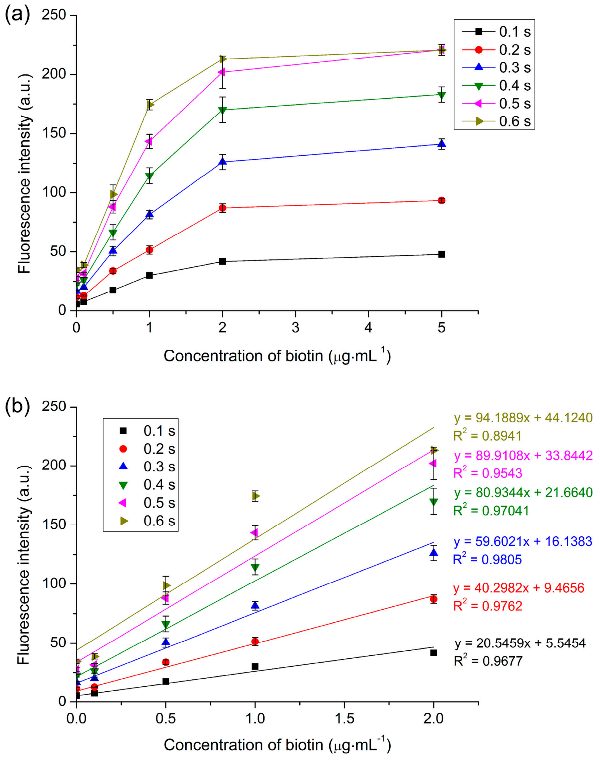


**Figure S1.** Optimisation of CCD camera exposure time. The fluorescence intensity of streptavidin functionalised particle plugs is plotted against the concentration of fluorescently labelled biotin at different CCD exposure times (0.1–0.6 s): (**a**) biotin concentrations of 0.1–5 μg·mL$^{-1}$, demonstrating a typical dose-response curve; and (**b**) linear range plotted for 0.1–2 μg·mL$^{-1}$ biotin concentration.

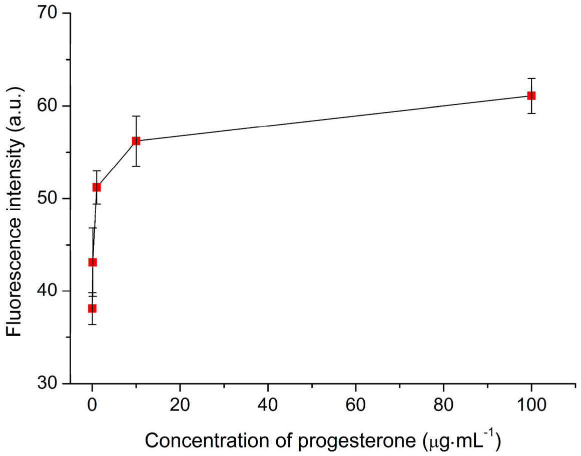


**Figure S2.** Fluorescently labelled progesterone (P4-FITC) assay. Magnetic particles functionalised with anti-P4 were exposed to different concentrations of P4-FITC and the resultant fluorescence intensities were measured. The plot follows a typical dose–response curve.
